# Predicting Agent Behaviour and State for Applications in a Roundabout-Scenario Autonomous Driving

**DOI:** 10.3390/s19194279

**Published:** 2019-10-02

**Authors:** Naveed Muhammad, Björn Åstrand

**Affiliations:** School of Information Technology, Halmstad University, Box 823, 30118 Halmstad, Sweden; bjorn.astrand@hh.se

**Keywords:** behaviour modelling, roundabout

## Abstract

As human drivers, we instinctively employ our understanding of other road users’ behaviour for enhanced efficiency of our drive and safety of the traffic. In recent years, different aspects of assisted and autonomous driving have gotten a lot of attention from the research and industrial community, including the aspects of behaviour modelling and prediction of future state. In this paper, we address the problem of modelling and predicting agent behaviour and state in a roundabout traffic scenario. We present three ways of modelling traffic in a roundabout based on: (i) the roundabout geometry; (ii) mean path taken by vehicles inside the roundabout; and (iii) a set of reference trajectories traversed by vehicles inside the roundabout. The roundabout models are compared in terms of exit-direction classification and state (i.e., position inside the roundabout) prediction of query vehicles inside the roundabout. The exit-direction classification and state prediction are based on a particle-filter classifier algorithm. The results show that the roundabout model based on set of reference trajectories is better suited for both the exit-direction and state prediction.

## 1. Introduction

Autonomous driving has gotten increased attention from the researchers and industrial community alike over the last 10–15 years. Even if the problem has long been investigated by researchers (demonstrated by examples such as ALVINN [1]), it was catalysed by the DARPA Grand and Urban challenges (held in 2005 and 2007, respectively) and resulting in many detailed research works (e.g., [2,3]). In addition to the advancement on algorithmic aspect of autonomous driving, these competitions also lead to the advancement on sensor side, with development of multi-beam lidar sensors such as the Velodyne range of devices [4] conceived and prototyped during the Grand Challenge [5]. Since then, a significant amount of research has been carried out addressing different aspects of autonomous driving such as object detection [6,7], localisation [8], tracking [9], intention estimation [10], as well as end-to-end deep-learning based approaches (e.g., [11]). In addition to the research explicitly addressing autonomous driving, a lot of closely-related research has also been carried out in the area of Advanced Driver Assistance Systems (ADAS); for example, Martinez et al. [12] investigated approaches for driving style recognition.

Traffic infrastructure and rules have evolved over a century, with human road user being the centre piece. While being in traffic scenarios ranging from residential neighbourhoods to motorways, we as humans continuously use the understanding of other humans’ behaviour, be it other drivers, bicyclists, pedestrians, etc., to keep ourselves and those sharing the roads with us safe. A detailed discussion on how our implicit understanding of behaviour, and subtle cues (e.g., eye contact, or the difference between a pedestrian talking to another near a curb, and then turning away and moving in the direction of road) are imperative for, or enhance, safety and efficiency in our everyday traffic is presented in [13].

In this paper, we focus on the task of entering a roundabout in the face of traffic already present inside (the roundabout). Roundabouts play a very important role in modern traffic infrastructure. Studies have shown that roundabouts reduce traffic accidents (in comparison to signal-controlled intersections), can reduce delays and improve traffic flows, and even have lower long-term cost compared to signal-controlled intersections [14]. As humans, we instinctively consider numerous factors such as the number of oncoming vehicles, their positions, speeds, even lengths, etc. to manoeuvre ourselves to enter roundabouts in a safe, efficient and smooth way. From an autonomous vehicles’ perspective, estimating agent (other vehicles present inside the roundabout) intention in terms of intended exit-direction, as well as agent’s future state in a roundabout, is not trivial but is imperative to make its manoeuvre to enter the roundabout safe, efficient (efficiency in this context not only includes time, but also smoothness of ride and minimisation of wear and tear to brakes, etc.) and smooth. We therefore, in this paper, investigate and propose methods for modelling traffic in roundabouts and predicting agent behaviour and future state (inside roundabouts). The proposed approaches are based on a setup where perception data is acquired by a lidar sensors located at the centre of a roundabout.

Modelling of behaviour and prediction of motion has long been of interest for the scientific community. It is applicable especially in domains where humans and intelligent systems co-exist [15], for instance service robotics, and assisted as well as autonomous driving. Other application areas of motion prediction include traffic monitoring, surveillance systems, etc. Consequently, comprehensive surveys have also been conducted on the subject. A survey which addresses motion-prediction applications in intelligent vehicles is presented in [16]. The survey is focused towards modelling approaches (mathematical models and algorithms, etc. that define how agent motion is modelled and how the predictions can be made) and proposes categorisation of these approaches into three main categories, namely physics-based, manoeuvre-based, and interaction-aware approaches. Physics-based approaches are relatively more parametric in nature, and are based on physical-models governing agent motion. Manoeuvre-based approaches consider motion patterns executed by agents while interaction-aware approaches also take into account interaction of manoeuvring entities with each other. The study argues that physics-based methods are computationally fast but are suitable for short-term predictions (typically not more than a second), whereas the manoeuvre-based and interaction-aware approaches allow for longer-term predictions but interaction-aware techniques are computationally expensive and thus not compatible with real-time operation requirements for applications such as risk-assessment in autonomous driving. Our method for motion modelling and state prediction is most closely related to the middle category i.e. manoeuvre-based as (which becomes apparent in Section 3) our method is based on comparing query trajectories to modelled or previously recorded real-world agent trajectories using a particle filter classifier.

Another comprehensive survey on motion-trajectory prediction that reviews over 170 works is presented in [15]. The study is more focused towards human motion but also considers bicycle and vehicles, and presents a systematic and deep (i.e., detailed) categorisation approach for motion-prediction methods. The authors of the study argued that the most basic level at which the works on motion prediction can be categorised is in terms of contextual cues and modelling approaches. They divide the modelling approaches into three main categories, i.e. physics based, pattern based and planning based. On a broader level, physics-based approaches model and predict motion based on physical-motion mathematical models. Pattern-based approaches exploit pattens in sequences of motion displayed by agents, and planning-based approaches go one step ahead by also taking into account longer-term goals of agents in motion prediction estimates. To make it more intuitively clear, Rudenko et al. [15] argued that these three categories of modelling approaches follow *sense-predict*, *sense-learn-predict*, and *sense-reason-predict* schemes, respectively. More interesting and novel however is the categorisation in terms of contextual cues proposed by Rudenko et al. [15]. The contextual cues include internal and external stimuli in relation to the agent in question, dynamics in the environment and properties of the static aspects of an environment itself. With respect to a target agent, the stimuli include position, orientation, velocity, and semantics such as age, gender, etc., whereas with respect to the environment presence of obstacles, map-awareness and affordances are considered, to name a few. In terms of contextual cues, our current study is based on motion state as we use heading angle, lateral offset and position as feature attributes. We also study the effect of taking vehicle size into account, while doing state prediction, thus our method has the ability to incorporate agent semantics into motion prediction. Our method is unaware of dynamics in the environment and assumes affordances to priority for vehicles (or an agent which is) already inside a roundabout with respect to any vehicles wanting to enter in future.

We describe our motion modelling and state prediction method in detail in Section 3, but before that, some related literature is presented in the following section.

## 2. Related Work

### 2.1. Manoeuvre-Based Motion Prediction

As introduced in the previous section, agent motion modelling and prediction approaches can primarily be divided into three categories [16]: (i) physics-based; (ii) manoeuvre-based; and (iii) nteraction-aware. Studies (e.g., [17,18]) that focus on acceleration and deceleration behaviour of different vehicle types employ physics-based models. Interaction aware approaches have not been as thoroughly investigated in the literature as the other two types, nevertheless Lefevre et al. [19] employed such techniques for instance for risk assessment in traffic. In terms of the manoeuvre-based approaches, Lefevre et al. [16] argued that such approaches are based on the assumption that, if the manoeuvre intention of a driver can be recognised early on, the future trajectory of the driver should match the manoeuvre. A central idea in such approaches is that real-world trajectories from roads can be clustered into groups, with each group representing a behaviour. The study also argues that, as a result of availability of digital maps, such real-world trajectories can sometimes just be extracted from structure of an environment. Based on a set of behaviours, manoeuvre-based motion prediction approaches employ estimation techniques, for instance Gaussian Processes [20,21] to then estimate most probable future manoeuvres. Recently, techniques such as deep-learning have also been applied to cluster vehicle encounters [22]. To compare a query and reference manoeuvres, manoeuvre-based techniques employ different distance metrics such as Hausdorff, Longest Common Subsequence, Euclidian distance, etc. [16]. Our own approach, proposed in the current paper, also falls under the manoeuvre-based approaches, but our focus is not limited to state prediction and also includes comparison between reference trajectories based on satellite imagery vs. real-word vehicle motion data.

### 2.2. Hierarchy of Driving Tasks from a Human Driver’s Perspective

Driving tasks can broadly be categorised as three levels of problems, i.e. strategic, tactical and operational [23,24]. The *strategic* tasks comprise of high-level (and longer-term) planning decisions such as route choice, cost estimates, etc., whereas *operational* tasks include low-level (short-term) and continuous routine tasks such as lateral control based on immediate environmental input. The *tactical* tasks fall in the middle of the two and are mid-level, medium-term tasks including, but not limited, to turning, overtaking, gap adjustment, merging, etc. [23].

Despite humans being efficient at all three levels of the driving tasks, the *tactical* tasks can nevertheless be more challenging for human drivers. For instance, a problem investigated thoroughly in the literature is the existence of a dilemma zone, where a human driver, when approaching a signallised (i.e., traffic-light controlled) intersection showing a yellow light, finds it difficult to decide whether to stop or pass through [25,26,27]. The time frame from this dilemma zone is 2.5–5.5 s before entering the intersection for most drivers [25].

### 2.3. Motion Prediction in Roundabout

As mentioned in Section 1, entering a roundabout smoothly and efficiently, despite being relatively trivial for humans, is not so simple from an autonomous vehicle perspective. The study of traffic in roundabouts, and motion prediction more specifically, has been studied in the literature, albeit not as intensively as t-junctions, intersections, etc.

A study that employs support vector machines to classify vehicles inside a roundabout to either stay or leave the roundabout is presented in [28]. Similarly, a study to estimate the effects of roundabout layout on driver behaviour, employing simulation data, is presented in [29]. A method for estimating reachable paths using conditional transition maps is presented in [30]. A study that employs a stereo camera setup for time-to-contact estimation is presented in [31]. The study is focused towards risk assessment instead of efficiency and smoothness of drive, when entering a roundabout.

In our current study, we investigate the *tactical* task of entering a roundabout from an autonomous vehicle perspective. In this context, we propose three ways of modelling agent motion inside a roundabout scenario. We then propose a method for employing these models for prediction of future state of agents, including exit-direction and position (for a given time frame), based on a particle filter classifier. We also compare the three roundabout models for the aforementioned tasks of exit-direction and state prediction. Finally, we also investigate how semantic information can be used to improve the state prediction.

## 3. Method

We propose a method for estimation of future position of an agent. The estimation is based on current agent state (position and speed) and its predicted future intentions. Below, we first describe three ways to model agent paths (or more specifically traffic flow in a roundabout in our context) in Section 3.1, proceeded by intention estimation (Section 3.2) and position prediction (Section 3.4) using these models.

### 3.1. Modelling Agent Paths—Case of a Roundabout

The three models for agent intention estimation and consequent position prediction are based on: (i) geometric-model of a roundabout; (ii) a data-driven mean model of the roundabout; and (iii) a model based on a set of reference trajectories traversed by agents at the roundabout.

Here, it is worth mentioning that the models (for behaviour modelling) and method (for state prediction) we propose in this study do not strictly depend on the choice of perception sensor employed to acquire vehicle motion data. Even if the current study employs an infrastructural perception sensor (a multi-beam lidar, to be more specific), our approach is based on vehicle motion information. In long-term future, such information might become available via telematics. However, full-scale deployment of autonomous vehicles is expected to take a very long time [13]. In the mean time, autonomous and connected vehicles are expected to share the roads with more conventional human-driven vehicles. During this transition period, infrastructural perception sensors can be installed in urban environments but their cost prohibits their widespread use. In this scenario, it is beneficial to study and compare different road-infrastructure (roundabout for the case at hand) models to compare the gain and loss in terms of generality, ease of building a model, etc. for applications such as behaviour modelling and state prediction. This is the rationale for the three models (based on roundabout geometry only, mean paths traversed by vehicles, and a set of actual vehicle trajectories, respectively) proposed as follows.

#### 3.1.1. Geometric Model of a Roundabout

The first proposed method to model a roundabout, and the most generalisable, is based solely on roundabout geometry, as also suggested in [16]. A large body of literature exists in the form of standards as well as research studies addressing types, geometric design, safety, speeds and other aspects related to roundabouts. For instance, authors in [32] describes different types of roundabouts, i.e., large, compact, double, etc. Roundabout safety, causes of accidents, and ways to mitigate them have been addressed in several studies (e.g., [33,34,35,36]). Such geometric models are easy to generate using, for instance, CAD drawings, satellite imagery, an image acquired using a drone, etc., and taking into account traffic flow direction, speed limits, etc. at the roundabout. This ease makes such models interesting to be investigated for applications in assisted and autonomous driving.

**Figure 1:**
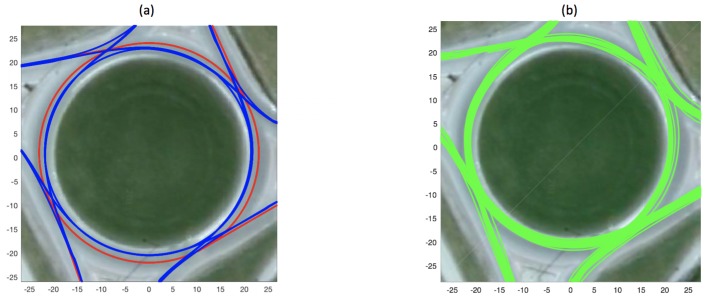
(**a**) Geometry (red) and mean of trajectories (blue) based models of the roundabout; and (**b**) model based on a set of reference trajectories. Each trajectory plotted in green represents a path taken by a real vehicle inside the roundabout (units in m).

The roundabout at which the data (explained in more detail in Section 3.5) for this study were acquired lies at the intersection of a university campus (with 30 km/h as speed limit) and a suburban zone (with 50 km/h as speed limit) and is a single-lane roundabout. Geometrical design characteristics such as minimum central island diameter and circulating carriageway width are presented in [37]. The study places the lowest acceptable central-island diameter and carriageway width for such speeds at 10 m and 7.6 m, respectively, and encourages larger central-island diameters for better safety. Similarly, a technical report by Federal Highway Administration (Washington, DC, USA) [38] demonstrates the optimal paths that drivers are expected to take in single-lane roundabouts, such as the one used to acquire data in our study. Keeping these studies in mind and the geometry of the roundabout at hand, models for trajectories excepted to be taken by vehicles entering and exiting at each of the four legs (16 cases in total) were constructed and are used as the first of the three models in our study. The model is presented as red trajectories in Figure 1a.

#### 3.1.2. Using a Set of Reference Trajectories

The second model that we propose to model agent paths inside a roundabout takes a *wisdom-of-the-crowd* approach and uses a set of *n* actual vehicle trajectories (traversed by vehicles inside the roundabout) for each of the 16 cases of vehicle entry/exit combination as the roundabout model. The model therefore is less generalisable compared to the geometric model presented above, as the trajectories recorded inside a single specific roundabout would represent vehicles’ behaviour only in that roundabout. This modelling approach was also used in our previous study [39]. A set of reference trajectories constituting such a roundabout model is presented in Figure 1b.

#### 3.1.3. Data-Driven Model of a Roundabout

The third model that we propose for a roundabout is neither explicitly based on the roundabout geometry nor on recorded trajectories themselves, but instead is based on mean paths taken by vehicles inside a roundabout. Such a model lies in the middle of the first two proposed models (i.e., geometric and set of reference trajectories) in terms of generality, and therefore also provides a middle ground for comparing the proposed models.

The data-driven model used in this study was constructed by recording vehicle trajectories over 40 min in the roundabout, and then calculating average paths taken by each of the 16 combinations of vehicle entry and exit location (i.e., for entry and exit at each of the four legs). The model is represented by blue trajectories in Figure 1a.

### 3.2. Classification of Exit Direction

Having one of the above three models at hand, as a new query trajectory begins to comes in, the first task is to predict what exit direction is the agent expected to take (to leave the roundabout) so that more precise predictions such as future agent state can be estimated. For the purpose of exit direction classification, we use and build upon our previous work [39], where we proposed exit direction classification using a decision-tree based as well as a particle-filtering based approach, using heading angle, lateral position offset and speed as feature attributes. The study showed that, in a multi-class problem such as the roundabout, the particle-filtering based approach performed better.

The work presented in [39] proposes exit-direction classification based on a set of reference trajectories and hence is comparable to the model presented in Section 3.1.2 above. This means that the number *n* of reference trajectories in the model is not fixed and can change depending on factors such as availability of data and complexity of problem at hand, and can usually range between a few tens to a few hundreds [39]. The fundamental difference, in terms of exit direction classification, when compared to the other two models presented in Section 3.1.1 and Section 3.1.3 is that in these two cases the reference trajectories in the model are fixed to one trajectory per class, i.e., a total of 16 trajectories in each model for the case of roundabout.

**Figure 2:**
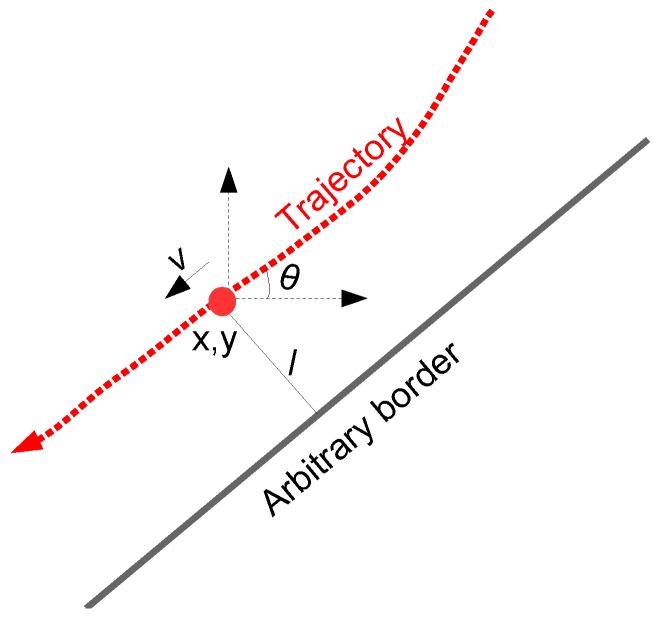
Feature attributes—θ, *l*, and *x, y*—used in the current study. The attribute *v*, which was found to be the least discriminative in our previous study [39], is not employed in the current study.

In our previous study [39], heading angle θ was found to be the most discriminative feature attribute and lateral position offset *l* was also found to perform well, whereas, speed *v* was the least discriminative feature attribute. For the sake of consistency with the findings of our previous study, we use {θ,l} as our primary feature set for the results presented in this paper. In the current study, we also investigate position (i.e., *x, y* coordinates) as a feature attribute (and briefly comment on it in Section 5.5). A depiction of the feature attributes is presented in Figure 2. It is worth mentioning here that the current study does not aim to compare different feature attributes, but instead focuses on modelling of roundabouts and employing such models in exit-direction classification and future state prediction for vehicles in a roundabout.

Using particle-filter based classification, the classifier output a probability of an incoming query trajectory to belong to each of the reference trajectories (i.e., 16 trajectories in case of the geometric and the data-driven, and *n* trajectories in the case of the set-of-reference-trajectories model) present in the roundabout models being used. The details on particle-filter classification method can be found in [39] but they are also summarised below for the sake of completion.

### 3.3. The Particle-Filter Classifier

**Figure 3:**
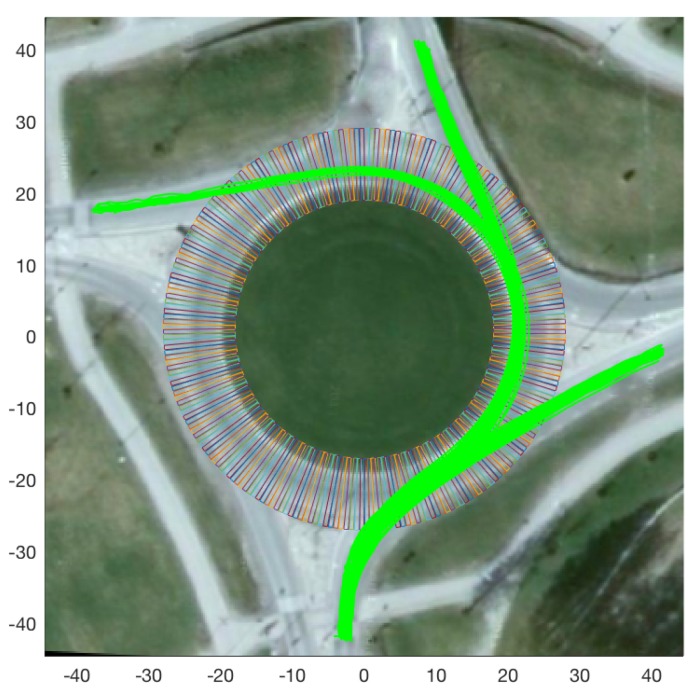
The grid discretising the physical space of interest at the roundabout (superimposed on sample trajectories originating from south and finishing in different directions).

As described in [39], we discretise an environment of interest (the roundabout in our case) using a grid of physical locations. Such a discretisation, for the case of the roundabout at hand, is shown in Figure 3, with each cell being 0.6 m wide (A discussion on the effect of larger grid-cell width is presented in [39]. In general, wider grid cells result in inferior performance by the classifier.) and 10 m in length. The behaviour model *B*, for any of the three roundabout models presented in Section 3.1, consists of the average feature attribute values (for each trajectory) inside each grid cell, and can be denoted as follows:

(1) B={θ,l,x,y∈IR}

The belief of a query trajectory to belong to the reference trajectories (for any of the three models), given the grid cells are described by *d*, is represented by the set of *M* number of particles $X_{d}$.

(2) $$X_{d}=\left\{x_{d}^{\left[1\right]},x_{d}^{\left[2\right]}..x_{d}^{\left[M\right]}\right\}$$

Each of the *M* number of particles, and its associated weight $w_{d}^{\left[m\right]}$, in the set $X_{d}$ represents the belief for the query trajectory to belong to one of the reference trajectories. At the beginning, a particle set $X_{0}$ is randomly generated with all available reference trajectories uniformly represented. Initial weights $w_{0}^{\left[m\right]}$ are all assigned equal values of 1/M.

For each change of location in terms of *d*, the particle set is then recursively updated as follows:

1. Make the observation $z_{d}$, i.e., measuring the feature attributes at current grid location of the query vehicle.
2. Calculate new weight for each particle depending on consistency between $z_{d}$ and the belief represented by the respective particle.
  (3) $$w_{d}^{m}=p\left(z_{d}|x_{d}^{m}\right)$$
3. Draw *m* particles, with replacement, employing updated particle weights.

At any given grid location in which a query agent falls at a given time, the sum of probabilities of particles representing reference trajectories in each of the possible (i.e., 16) categories represents the belief of the query instance to belong to that category.

### 3.4. State Prediction in Tactical Timeframe

As mentioned in Section 1, even for human drivers, when approaching a traffic light, a dilemma zone exists between 2.5 and 5.5 s before arriving at the traffic light, where it is difficult for a driver to decide between stopping or not, when seeing a yellow light [25]. In the context of the current study, *tactical* timeframe refers to such a timeframe and we denote it by $t_{tac}$.

Given a tactical time window $t_{tac}$ and current speed $v_{curr}$ of a query vehicle, the state prediction amounts to estimating the position of the vehicle $t_{tac}$ into the future. For the geometric and data-driven roundabout models (cf. Section 3.1.1 and Section 3.1.3), the state prediction is done by traveling along the corresponding reference trajectory (of the winning class, out of the total 16) with a uniform speed value $v_{curr}$ for time $t_{tac}$. In the case of the set-of-reference-trajectories roundabout model (cf. Section 3.1.2), as each of the 16 classes is represented by multiple reference trajectories, a position is predicted in the same manner $t_{tac}$ s into the future (for each reference trajectory represented in the particle set), and then a weighted average is performed on the predicted position estimates to arrive at a consolidated future position estimate. In this way, in the set-of-reference-trajectories model, general speed and acceleration patterns demonstrated by vehicles in the roundabout are taken into account in state prediction implicitly.

**Figure 4:**
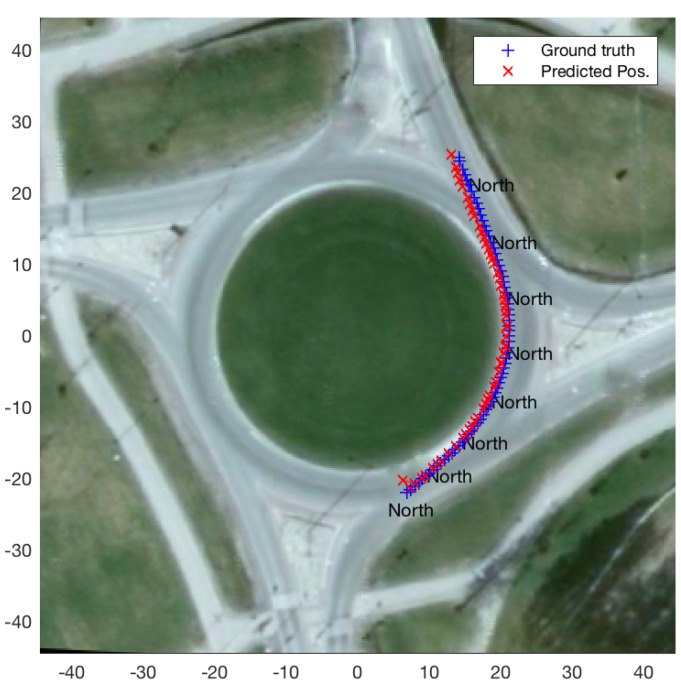
Exit direction classification and position prediction for a query trajectory inside a roundabout, using roundabout model based on a set of reference trajectories.

Figure 4 depicts a demonstrative experiment for exit-direction classification (described above in Section 3.2) as well as state prediction, for a single query vehicle trajectory at a roundabout (for $t_{tac}$ = 1 s). The figure shows the predicted exit direction (shown in the figure once every ten estimates, for clarity) as well as the ground-truth and predicted positions while the query vehicle remains inside the roundabout. It is worth mentioning here that, for this experiment, the roundabout model used is the one based on a set of reference trajectories (cf. Section 3.1).

### 3.5. Experimental Setup

Below, we describe details about the dataset used for experimentation as well as different experiments performed to compare the proposed roundabout models, validate the corresponding state prediction, etc.

#### 3.5.1. Dataset

The dataset used for experimentation consists of real-world traffic data, acquired at a busy roundabout that has four legs, and a central-island diameter of 40 m. The dataset was acquired by Kucner et al. [30] and Fan et al. [40] by placing a Velodyne HDL-64E multi-beam lidar sensor at centre of the roundabout for 2 h and scanning at 10 Hz (i.e., ten 360${}^{\circ }$ scans per second), in Sweden (the traffic flow is therefore *right-hand*.) The dataset consists of 1694 vehicle trajectories, extracted from the raw point-cloud data captured by the lidar sensor. Sample trajectories from the dataset are shown in Figure 1b. The dataset contains different vehicle types. We categorised the vehicles based on their length (for vehicle-category based experiments described later in Section 3.5.5) into five categories namely:

- small vehicles (below 2.5 m) such as motorcycles;
- cars (between 2.5 and 6.3 m), including not only cars but also SUVs, etc.;
- large vehicles (between 6.3 and 8 m) such as vans/mini-busses;
- trucks (between 8 and 12 m) e.g. trucks typically in a single body; and
- long vehicles (above 12 m) typically consisting of a prime mover and one or more trailers.

**Figure 5:**
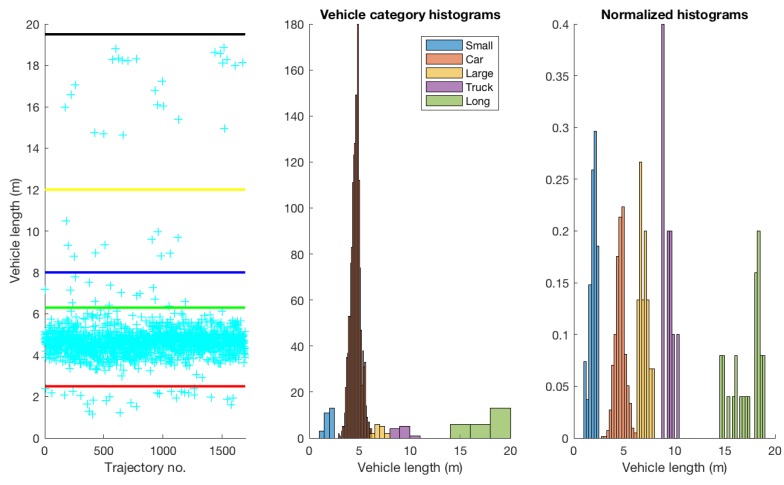
Vehicles categorised by length into five categories, namely small, car, large, truck and long vehicles: (**a**) each instance (unique vehicle in the dataset) is represented by a cyan cross with the upper boundaries for the smallest to longest categories represented by the red, green, blue, yellow and black lines, respectively; (**b**) histograms for each length-based vehicle category; and (**c**) normalised histograms.

Figure 5 shows the distribution of different vehicle types in the dataset. Not surprisingly, the majority of vehicles in the dataset belong to the car category, with 1617 vehicles (out of the total 1694 in the dataset). Other categories contained much fewer vehicles: 27 small, 15 large, 10 truck, and 25 long vehicles. A question might arise here as to why were the vehicles in the large and truck categories considered as two separate categories and not a single one. The reason for this is that van busses and light cargo vehicles are physically different compared to heavy single-body trucks (and are therefore expected to behave differently on road).

#### 3.5.2. Evaluation of Exit Direction and State Prediction Results

We report the result of exit-direction classification in terms how early in time, before a query vehicle takes its ground-truth exit, the exit prediction by the proposed algorithms converged (to the correct exit) and stayed robust. The reason for using exit time as reference for exit-direction classification is twofold. Firstly, in terms of tactical decision making, time leading to a final event is of importance (for instance, in the case of a dilemma zone at traffic lights [25], as mentioned in Section 1). Secondly, the lengths of vehicle trajectories in the roundabout use case at hand differ significantly in case a vehicle (regardless of entry direction) takes the first, second or third corresponding exit. Therefore, time, taking vehicle exit from the roundabout as reference, serves as a more meaningful measure of correct exit-direction classification, compared to, for instance, time or distance traveled measured from vehicle entry into the roundabout. Here, it is worth mentioning that our previous work [39] used distance (from convergence to exit from the roundabout) as a measure of exit-direction classification performance.

The state prediction evaluation results are presented in terms of mean absolute Euclidean distance error between predicted ($t_{tac}$ into) future positions and the ground-truth position that the vehicle actually arrived at $t_{tac}$ into future.

Here, it is worth mentioning that for the exit-classification and state-prediction results presented in upcoming Section 4.1 and Section 4.2, 15% (i.e., 254) of the 1694 trajectories were randomly chosen as reference trajectories and the remaining (1440) were used for validation. Seeding for the random choice of the 15% reference trajectories was kept fixed for the sake of consistency between experiments.

#### 3.5.3. Comparison of the Three Roundabout Models

The three roundabout models presented in Section 3.1 are compared with each other in terms of how well each model can be employed for exit-direction classification and state prediction using the (time since convergence, and position error) measures described above in Section 3.5.2.

#### 3.5.4. Tactical Time Widows

Instead of limiting to a single value of $t_{tac}$ to compare the three roundabout models, results are reported for the following tactical time windows:

(4) $$t_{tac}\in \left\{1s,2s,3s\right\}$$

Here, it is worthing mentioning that, while it would be interesting to experiment with longer values of $t_{tac}$, the roundabout at hand is too small to provide sufficient data points for meaningfully investigating larger $t_{tac}$ values at all relative exits (i.e., first, second or third exit, regardless of the entry direction).

#### 3.5.5. Tactical Prediction Based on Vehicle Type

Intuitively, one can argue that inside traffic, different-sized vehicles would behave differently. A motorcyclist will manoeuvre his/her vehicle differently compared to an SUV, or compared to a long vehicle consisting of a prime mover and multiple trailers, for instance. We report results on state prediction using different vehicle categories (cf. Section 3.5.1) as separate, as well as when considering them as just one general group.

## 4. Results

The results for exit-direction classification, and state prediction (overall as well as based on vehicle categories) are presented in this section.

### 4.1. Exit-Direction Classification

As devised in Section 3.5.2, the results for exit-direction classification in terms of time since convergence (taking exit from the roundabout as reference) are presented in Table 1. The three rows of the table correspond to the three ways of modelling agent paths in the roundabout (cf. Section 3.1) and the three columns represent the traffic taking first, second and third exit, respectively, at the roundabout (regardless of actual entry or exit direction at the roundabout).

**Figure 6:**
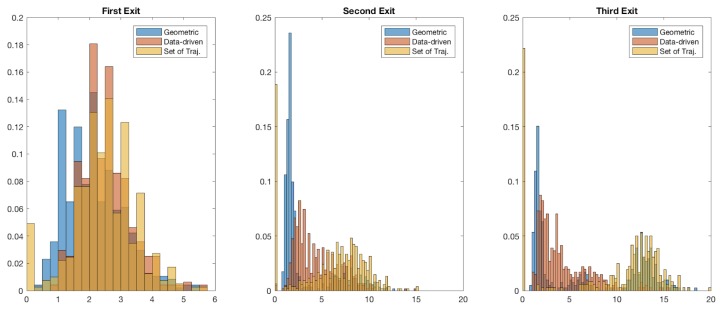
Exit-direction classification. Normalised histograms for time since convergence: (**a**) first exit; (**b**) second exit; and (**c**) third exit. Note that the bin-size used for all the histograms is uniform, but the axes have been left non-uniform for clarity.

Figure 6 presents a deeper insight into the results of Table 1. The figure presents normalised histograms of time since convergence for the first, second and third relative exits (Figure 6a–c, respectively) for each of the three roundabout models.

### 4.2. State Prediction

Results for state prediction in terms of mean absolute Euclidean distance error between predicted and ground-truth positions ($t_{tac}\in \left\{1s,2s,3s\right\}$ into the future) are presented in Table 2.

**Figure 7:**
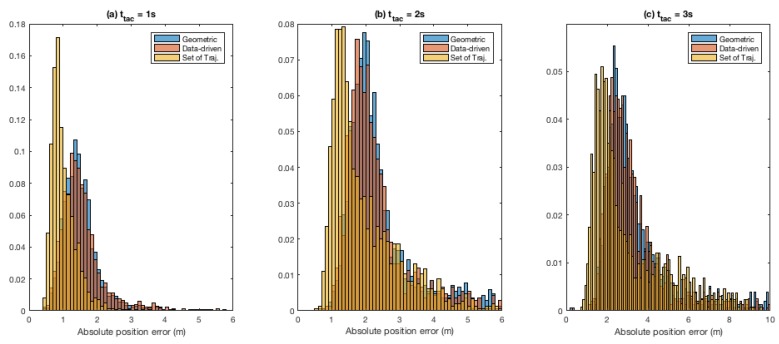
State prediction. Normalised histograms for mean absolute position error: (**a**) prediction time $t_{tac}$ = 1 s; (**b**) $t_{tac}$ = 2 s; and (**c**) $t_{tac}$ = 3 s. Note that the bin-size used for all the histograms is uniform, but for the sake of clarity the axes have been left non-uniform, and the histograms in (**b**,**c**, have been truncated at 6 m and 10 m, respectively.

Figure 7 presents normalised histograms for absolute mean position errors for $t_{tac}\in \left\{1s,2s,3s\right\}$ (in Figure 7a–c, respectively) for the three roundabout models. Bin size used in all the histograms presented in the figure is uniform but axes have not been equalised and histogram tails in Figure 7b,c have been truncated (at 6 m and 10 m, respectively, resulting in omission of some outliers) for clarity.

### 4.3. Tactical Prediction Based on Vehicle Type

Mean absolute Euclidean distance error between predicted and ground-truth positions for different vehicle categories (cf. Section 3.5.1) is presented in Table 3. The method used for modelling agent paths in the roundabout for the results presented here is that of Section 3.1.2, i.e. using a set of reference trajectories as the model.

The results presented in the first row of the table for the instance where all the vehicles are considered as one class differ from the results in Table 2 in that, here, as the overall results are to be compared with category-wise results, the number of data points from each category contributing in this *overall* experiment are balanced. In other words, as the aim of this experiment is to compare categorisation of vehicles into different classes versus treating them as a single class, the same number of data points from each category were used in the *overall* test to avoid bias towards the cars category which otherwise has the largest number of data points (cf. Section 3.5.1—cars constitute 1617 of the total 1694 trajectories in the dataset).

## 5. Discussion

### 5.1. Comparing the Roundabout Models

#### 5.1.1. In Terms of Exit-Direction Classification

In terms of exit-direction classification, as Table 1 depicts, the roundabout model based on a set of reference trajectories (cf. Section 3.1.2) seems to perform the best, compared to both the other models, i.e. the geometric and the data-driven model (of Section 3.1.1 and Section 3.1.3, respectively). The table shows that the exit-classification method converges and stays robust much earlier in the case of using set of reference trajectories as roundabout model, compared to the other two models. This difference is especially pronounced in the case of second and third exits. Intuitively, when the three models are compared in terms of their generality, the geometric model is the most generic representation of the roundabout, whereas using a set of reference trajectories as the roundabout model is the most specific one can get to the specific traffic infrastructure location (in our case, the roundabout at hand). Therefore, it is unsurprising that using set of reference trajectories as roundabout model performs the best in this situation.

Figure 6a shows that in the case of first relative exit, the three models have similar overall spread. Figure 6b,c, however, indicates that, for the case of second and third exit, the model based on set of reference trajectories performs better (i.e., longer time-since-convergence values) compared to the other two models. As described in Section 3.5.2, our metric of *time since convergence* is based on time since convergence and staying robust (to ground-truth exit direction) until exit from the roundabout. The high peaks near 0 s include instances where even just one erroneous exit-direction classification was made when the query vehicle was nearing exit. While this metric gives a clear insight into the classifier, this behaviour can be remedied using smoothing (for instance using a voting scheme) for exit-direction predictions made by the classifier.

#### 5.1.2. In Terms of State Prediction

In terms of mean Euclidean distance error between the predicted and ground-truth future positions (cf. Table 2), as one would expect, the error grows with prediction time regardless of the model used. When the three models are compared, the roundabout model based on set of reference trajectories again performs better than the other two models. This is evident also in Figure 7, which shows that for all the three $t_{tac}$ values, histogram for set-of-reference-trajectories model clearly lies earlier (i.e., lower mean absolute position errors, overall). This difference between the models becomes more pronounced as $t_{tac}$ increases from 1 to 2 and 3 s.

#### 5.1.3. Effect of Small Number of Reference Trajectories

**Figure 8:**
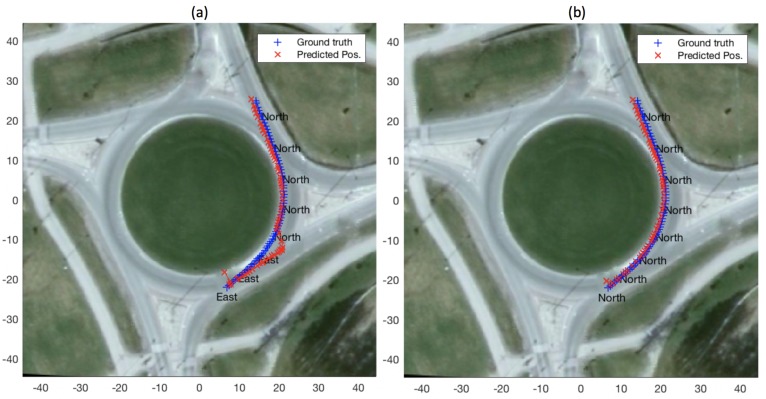
Exit-direction classification and state prediction using a set of reference trajectories as roundabout model: (**a**) employing 16 reference trajectories in the model; and (**b**) employing 100 reference in the model.

Better results, in terms of both exit classification and state prediction, also owe to the difference in the number of unique reference datapoint available to our filtering based classification and prediction methods. In the case of geometric and data-driven roundabout models, the number of unique reference trajectories available to the filter are 16 i.e., one of each of the 16 pairs of entry and exit directions. A particle filter running with *p* number of particles will essentially start with replications of the 16 reference trajectories. In the case of the roundabout model based on set of reference trajectories the number of reference trajectories is only limited by the number of data points collected during the data acquisition phase. Figure 8 shows exit-direction classification and position prediction results for an example query trajectory, for the cases where set-of-reference-trajectories model comprised of 16 reference trajectories in one case and 100 in the other (it is worth mentioning that in both cases *p* was set to 1000). The figure shows that in the case of 16 reference trajectories the filter takes much longer to switch from convergence to the east exit to the ground-truth north exit, compared to the case where 100 reference trajectories are employed.

### 5.2. State Prediction Using All vs. Exit-Direction Reference Trajectories

**Figure 9:**
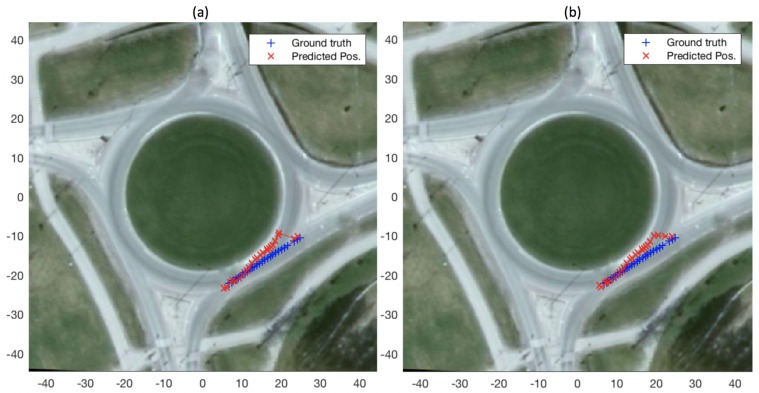
Comparison in state prediction when all reference trajectories are used (**b**); and when only the reference trajectories belonging to the winning (i.e., belonging to the estimated exit-direction at any given time) class are used (**a**).

For estimating the future location of an agent, as described in Section 3.4, one intuitive option is to only use the reference trajectories (from the whole set of reference trajectories represented by the particle set in the particle-filter classifier) belonging to the class that is predicted as the exit direction at any given time. In practice, however, we found that it results in loss of generality compared to when all the reference trajectories represented by the particle set at a given time are considered for state prediction. A demonstration of this is presented in Figure 9, which shows that the evolution of predicted position is more smooth when using all the reference trajectories represented by the particle set (Figure 9b) versus when only those of the exit-direction class are used (Figure 9a).

### 5.3. Merits of Categorising Vehicles

Categorising vehicles into multiple classes has multiple aspects of interest from the point of view of agent behaviour modelling in traffic. In the case the available perception system allows for the detection of vehicle class, such a categorisation can enhance predictions about any future behaviour of the agent/vehicle. Conversely, if the behaviour of different categories of vehicles in traffic is significantly different, then recognition of behaviours may lead to identifying vehicle class without the need of explicitly doing it using the perception system at hand. Our experiments reported in Table 3 targeted the former goal, i.e. getting insight into the merit of using vehicle class for improving state prediction. As the table shows, overall, for the three time windows, the error in position prediction improves (in comparison to considering the traffic as one single class) for half of the instances, especially for the categories of car, truck and long vehicles. On the other hand, in the other half of the instances, and especially for the small and large vehicle classes, the error gets worse.

One reason for improvement of prediction error for some vehicle classes and its deterioration for others can be the nature of the classes. As mentioned in Section 3.5.1, the categorisation of vehicles was done based primarily on vehicle length. It is possible that the inconsistent (i.e., in terms of improvement in prediction error) classes might have sub-classes within them.

**Figure 10:**
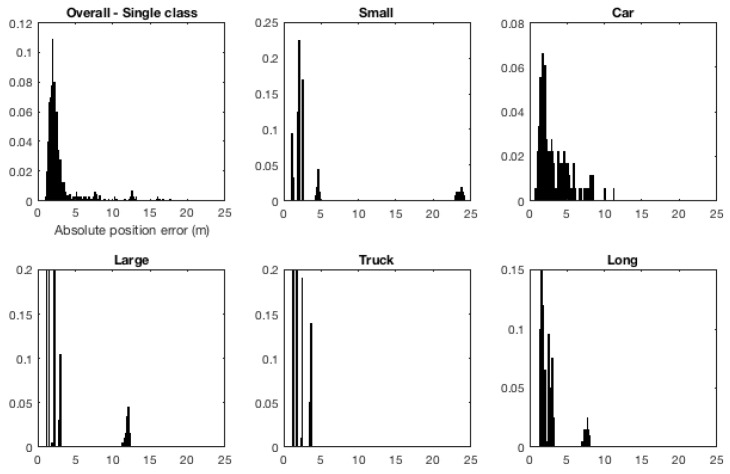
Normalised histograms for mean absolute position errors when considering all vehicles (traversing the roundabout having a central-island diameter of 40 m) as a single class versus categorising them into small, car, large, truck and long vehicles (for $t_{tac}$ = 3 s). Bin size for all the histograms is uniform. The y-axis has been left non-uniform for clarity.

In general, given the changes in prediction error when categorising traffic into classes indicates that such a categorisation is significant in terms of agent behaviour modelling for state prediction in traffic. Looking into histograms of absolute mean position errors also provides a similar insight. Figure 10 shows normalised histograms for absolute mean position errors for the tests reported in Table 3 for $t_{tac}$ = 3 s. It is worth mentioning here that the histograms look less *continuous* compared to the histograms in Figure 7 because there are much fewer data points available for performing vehicle-type based tests for some classes in comparison to the whole dataset (cf. Section 3.5.1). It can be noted from the figure (i.e., Figure 10) that the histogram when considering all vehicles as a single class has a long tail, similar to that in Figure 7c. The tails become much shorter (with lower maximum errors) when categorising vehicles into classes. This is especially the case for the categories of car, truck and long vehicles. One reason for this pattern is that when all vehicles are considered as a single class, a query vehicle belonging to for example Long class would be compared with reference trajectories belonging a mix of classes, resulting in a less precise state estimate. On the other hand, when categorising vehicles into classes, reference trajectories are likely to more closely resemble the behaviour of a query trajectory at hand, resulting in improved performance of state prediction.

### 5.4. Cause of High Error in Position Prediction

**Figure 11:**
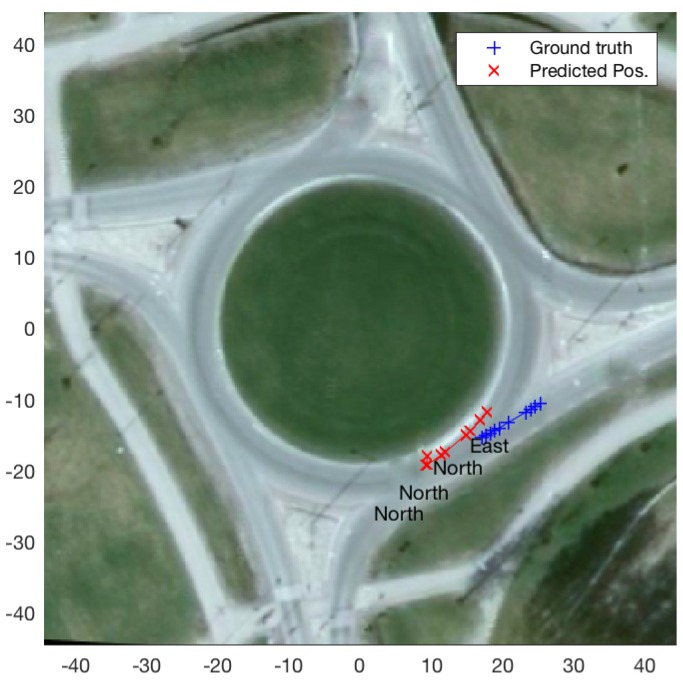
An instance with high mean error of 8.04 m. Primary cause for high error in state prediction is erroneous exit-direction estimate.

As apparent in Figure 7 and Figure 10, mean position error in some cases is high, especially as $t_{tac}$ increases. The reason for high mean position error is misclassification of vehicle exit direction. Figure 11 shows one instance where state estimation for $t_{tac}=3$, using set of reference trajectories as the model, results in a mean position error of 8.05 m. Misclassification of exit direction tends to occur (and intuitively so) in the beginning (i.e., just after the entry of a query vehicle into the roundabout) and results in incorrect (i.e., belonging to other exit directions) reference trajectories to be employed for state prediction.

### 5.5. Other Feature Attributes

In addition to the primary feature set {θ,l} used in this study, we also tried other feature sets comprising of different combinations of feature attributes shown in Figure 2 (excluding the speed *v*, as our previous study [39] had found it to be a less discriminative attribute). In general we found that feature set {θ,x,y} gives very similar results to those using {θ,l}. On the other hand, the results degrade when only {x,y} is used. This is apparent in Table 4 and Table 5, which show mean-time-since-convergence metric for the feature sets {x,y} and {θ,x,y}, respectively.

Comparing Table 1 and Table 4 also reveals the degradation in performance when using {x,y} as feature set. Table 4 shows that the performance of the geometric and data-driven models does not change significantly regardless of whether a query vehicle is taking the first, second, or third exit. In other words, the two models only converge and stay robust very close to the ground-truth exit of a query vehicle. On the other hand, in the case of roundabout model based on set of reference trajectories, we see that the time of staying robust after convergence increases for query vehicles taking the first, second or third exits, as one would logically expect. In contrast, when {θ,l} is employed as feature set, the time since convergence increases from first to second and third exit, for all the three models.

Mean position error results for feature sets {x,y} and {θ,x,y} showed a similar trend in comparison to the primary feature set {θ,l} and are therefore not included here for the sake of brevity.

**Figure 12:**
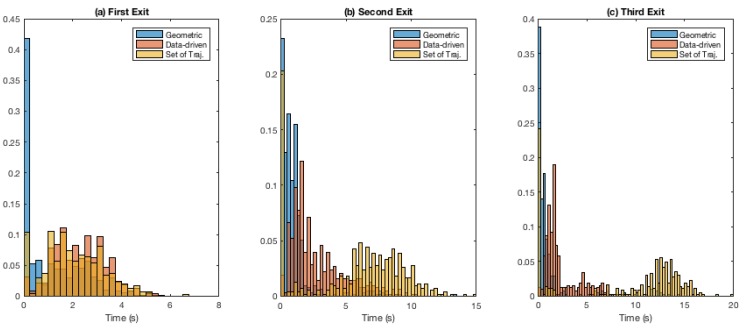
Exit-direction classification using feature set {x,y}. Normalised histograms for time since convergence: (**a**) first exit; (**b**) second exit; and (**c**) third exit. Note that the bin-size used for all the histograms is uniform, but the axes have been left non-uniform for clarity.

The degradation in results when employing {x,y} as feature set in comparison {θ,l} can also be observed in Figure 12, which presents normalised histograms of time since convergence for the first, second and third relative exits for each of the three roundabout models, using feature set {x,y}. In comparison to Figure 6, Figure 12 shows increased instances close to 0 s in the case of geometric model, revealing deterioration in the performance of geometric model when used with the feature set {x,y}. (Please note the difference in axes limits when comparing the two figures. The axes have not been equalised for better viewing.)

## 6. Conclusions

This paper presents methods for on-road agent behaviour prediction and state estimation in the context of roundabout traffic scenario. The agent behaviour prediction is in terms of classifying which exit direction an agent inside the roundabout is expected to take, and the state estimation refers to the predicted future position of an agent after a given time. The paper also presents three models to model traffic inside a roundabout and compares them in terms of both the exit-direction classification and the state prediction. Among the three models, i.e., geometric (which can be generated using drawings or satellite images, etc.), data-driven, and based on a set of reference trajectories, experimental results indicate that the model based on a set of reference trajectories is better suited, in terms of both the early and robust exit-direction classification and more accurate state prediction. Experiments carried out in this study for state prediction by categorising vehicles into classes based on vehicle size indicate that such a categorisation can effect, and in some cases enhance, the state prediction accuracy.

In the future, we intend to investigate recognition of vehicle category based on behaviour only, and study the usage of deep learning for agent behaviour recognition in comparison to the filtering-based classifier employed in the current study. We also plan to use the methods proposed in the current study to develop a simulation where autonomous vehicles adjusts their manoeuvres in the face of real on-coming traffic in a roundabout and compare it to human behaviour (i.e., recorded human manoeuvres in the same situation).

## Figures and Tables

**Table 1:** Mean time (larger is better) from convergence to the correct exit direction until exiting from the roundabout.

	Rounabout Exit	First	Second	Third
Model Type				
Geometric		2.14 s	3.28 s	6.63 s
Data-driven		2.50 s	4.43 s	4.14 s
Set of ref. trajs.		2.44 s	5.99 s	9.42 s

**Table 2:** Mean error (smaller is better) in state prediction in tactical timeframe.

	Time *t_tac_*	1 s	2 s	3 s
Model Type				
Geometric		1.52 m	2.62 m	4.16 m
Data-driven		1.54 m	2.65 m	4.21 m
Set of ref. trajs.		1.14 m	1.99 m	3.11 m

**Table 3:** Taking vehicle type into consideration for state prediction in tactical timeframe in comparison to considering all vehicle types as a single class.

Vehicle Class	Mean Pos. Err. for $t_{\mathrm{tac}}=1s$	Mean Pos. Err. for $t_{\mathrm{tac}}=2s$	Mean Pos. Err. for $t_{\mathrm{tac}}=3s$
Overall (single class)	1.22 m	2.12 m	2.74 m
Small	2.52 m	4.85 m	5.05 m
Car	1.02 m	2.05 m	3.15 m
Large	1.87 m	2.73 m	3.92 m
Truck	1.39 m	1.68 m	2.17 m
Long	1.13 m	1.76 m	2.64 m

**Table 4:** Mean time (larger is better) from convergence to the correct exit direction until exiting from the roundabout.

	Rounabout Exit	First	Second	Third
Model Type				
Geometric		1.13 s	1.03 s	1.13 s
Data-driven		2.28 s	2.78 s	2.33 s
Set of ref. trajs.		2.04 s	5.78 s	8.95 s

**Table 5:** Mean time (larger is better) from convergence to the correct exit direction until exiting from the roundabout.

	Rounabout Exit	First	Second	Third
Model Type				
Geometric		2.13 s	3.26 s	6.62 s
Data-driven		2.50 s	4.43 s	4.19 s
Set of ref. trajs.		2.43 s	6.02 s	9.45 s
